# Prognostic Significance of NRAS Gene Mutations in Children with Acute Myelogenous Leukemia

**DOI:** 10.4084/MJHID.2011.055

**Published:** 2011-11-28

**Authors:** Rabab M. Aly, Mohamed R. El-sharnoby, Adel A. Hagag

**Affiliations:** 1 Clinical Pathology Department, Faculty of Medicine, Mansoura University, Egypt; 2 Pediatrics Department, Faculty of Medicine, Tanta University, Egypt

## Abstract

**Background:**

NRAS mutations are the most commonly detected molecular abnormalities in hematologic malignancies, especially in those of myeloid origin.

**Objective:**

We aimed to determine the frequency of NRAS (NRAS*^mutant^*) mutation; and its prognostic significance in Egyptian children with acute myelogenous leukemia (AML).

**Subject and methods:**

Peripheral blood and bone marrow (BM) samples were taken from 39 de novo pediatric AML patients. Twenty subjects with matched age and sex were selected as a control group. Samples from patients and control were analyzed for Exons 1, 2 of NRAS gene using genomic PCR-SSCP method.

**Results:**

NRAS mutations at the time of diagnosis was found in 6/39 (15.4%) AML cases. Patients with NRAS*^mutant^* had no significant improved clinical outcome than patients without mutation. Patients with NRAS*^mutant^* had similar complete remission (CR) rates compared with non-mutated patients (66.7% vs. 69.5%, P=0.43). Those in CR had a similar relapse rate regardless of the presence of NRAS*^mutant^* (RR 33.4% vs. 30.2%, P=0.26). However, an adverse prognosis for 3 year overall survival (OS) was associated with the presence of NRAS mutations. This adverse prognosis associated with NRAS mutations was also observed in terms of disease-free survival (DFS) (P=0.007). Univariate analysis showed that unfavorable prognostic factors for DFS were cytogenetic data (P = 0.005) and the NRAS gene mutation (P = 0.002).

**Conclusion:**

NRAS*^mutant^* did not contribute to increase the disease recurrence, however NRAS*^mutant^* was found to be a poor prognostic factor for children with AML. Further studies to confirm these findings are required because of the small number of patients with NRAS mutation.

## Introduction

Acute myeloid leukemia (AML) is suppression of normal hematopoiesis. Cytogenetic and characterized by expansion of myeloid blasts with molecular studies have defined AML as a heterogeneous disease.^1^ The presence of defined karyotypes is among the most important prognostic factors in acute myeloid leukemia (AML). However, even within defined cytogenetic groups stability of remission and long-term survival may vary significantly. Therefore, additional recurrent aberrations may have a prognostic impact.^2^ It has been showed that Pediatric AML patients may harbor more than one mutation at diagnosis, some of which with a possible prognostic impact.^3^–^7^ Mutations in the NRAS gene are one of these genetic aberrations that play a role in myeloid neoplasia.^8^

NRAS gene plays an important role in the regulatory processes that govern proliferation, differentiation and apoptosis;^9^ abnormality in this gene has been implicated in the pathogenesis of AML. RAS oncogenes encode a family of membrane-associated proteins, which regulate signal transduction upon binding to a variety of membrane receptors.^10^ There are three functional RAS genes (NRAS, KRAS and HRAS); in AML. K-RAS mutation occurs to a lower but still significant frequence in pediatric AML.^11^ NRAS is the most prominent; reported in 11%–30% of patients.^12^ All homologs were exclusively in codons 12, 13, and 61 conferring constitutive activation of the RAS protein, which is subsequently held in the GTP bound status leading to an increased activity of the RAS pathway causing an increased proliferation and a decreased apoptosis rate.^13^ RAS mutations were described in the various solid tumors as well as in hematologic malignancies. The prognostic impact of NRAS mutations is still under research and seems to vary from disease to disease,^14^ several studies indicated a poor prognostic impact for that mutation,^14^,^15^ and however Lapillonne et al confirmed this finding in pediatric AML.^16^ On the contrary, Neubauer et al found a favorable outcome for malignancies with NRAS mutations,^12^ and some studies failed to define any prognostic impact for NRAS mutations.^10^,^13^,^17^

We undertook this study to determine the frequency of NRAS mutation and its prognostic significance in a number of Egyptian pediatric patients with AML.

## Patients and Methods

Newly diagnosed pediatric AML patients were included in this study; cases were recruited from pediatric hematology clinics of Tanta University Hospitals, Tanta, Egypt. Peripheral blood and BM samples were obtained from 39 de novo AML cases at initial diagnosis obtaining an informed consent from patients or their guardians; they were 21 boys and 18 girls. The median age was 7.4 years (range, 5.6–13 years). The median percentage of blasts in the fresh bone marrow samples was (65%). All included patients were receiving the same treatment protocol approved by the Oncology Team of Tanta University Hospital TUH; in brief they received 1–2 cycles of 14–21 days of intensively timed induction chemotherapy (doxorubicin, Ara-C, 6-thioguanine and methotrexate) depending upon BM aspiration done at the end of each induction course. Additional consolidation regimens included 1–2 cycles of 12-days (doxorubicin, Ara-C, VP-16 and methotrexate). Then patients who did not achieve remission received intermittent chemotherapy (Ara-C, 6-thioguanine and methotrexate) every 3 months for 6 cycles with the standard follow-up care and regular BM aspiration every 21 days to confirm remission; *complete remission* means normocellular bone marrow contain less than 5% blast cells and showing evidence of normal maturation of other bone marrow elements as evidenced by the repeated BM aspiration. The average duration of follow up was mean ± SD (32 ± 2.24 months).

Patients were classified according to the standard methods; morphological according FAB classification, cytochemical and immunological evaluation.^13^ Informed consent was obtained from twenty subjects with matched age and sex who were selected as a control group. Samples from patients and control were analyzed for mutation in Exons 1, 2 of the NRAS gene using genomic PCR method.

## Cytogenetic Analysis

Cytogenetic investigations were performed by karyotyping G-banding analysis in all patients.^18^

## PCR of NRAS Gene

Genomic DNA was extracted from diagnostic bone marrow specimens of patients and control using the QIAamp DNA blood mini kit for DNA extraction provided by QIAGEN (Inc Chasworthy, CA). The concentration of extracted DNA was then measured by UV spectrophotometry at 260 & 280 nm and analyzed by electrophoresis on 2% agarose gel for detection of purity.

Separate assays were developed for mutation detection at (hot spots) in codons 12/13 (exon 1) and codon 61 (exon 2). Oligonucleotide primers amplifying short fragments {241 base pair (bp) for exon 1 and 201 bp for exon 2} were designed for PCR as follows:

### N12/13 assay

forward, 5′-GACTGAGTACAAACTGGTGG-3′; and reverse, 5′-TGCATAACTGAATGTATACCC-3′.

### N61 assay

forward, 5′ CAAGTGGTTATAGATGGTGAAACC-3′; and reverse, 5′-AAGATCATCCTTTCAGAGAAAATAAT-3′.

PCR amplification was performed in 50 ul reaction, contained 50–100 ng of genomic DNA, 10 mM Tris HCl (pH 8.3), 50 mM KCl and 1.5 mM Mg Cl2, 200 uM of each deoxyribonucleotide triphosphate (dNTP), 2.5 units Taq polymerase, and 6% dimethylsulphoxide. PCR conditions were as follows: ***(1) Exon 1***, HotStarTaq (Qiagen, Valencia, CA), and 0.625 U. Primers (12.5 pmol), N12/13 forward, N12/13 reverse. Denaturing, 95 C for 15 minutes and 94°C for 30 seconds; annealing, 55.5°C for 1minute; extension, 72°C for 1 minute for 35 cycles; final cycle at 72°C for 10 minutes. ***(2) Exon 2***, HotStarTaq (Qiagen), 0.625 U. Primers (12.5 pmol), N61F forward, N61R reverse. Denaturing, 95°C for 15 minutes; 94°C for 30 seconds; annealing, 55.5°C for 1 minute; extension, 72°C for 1 minute for 35 cycles; final cycle at 72°C for 10 minutes.

**Figure 1:**
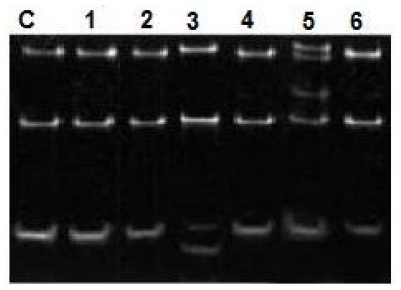
SSCP of PCR products from NRAS exon 1. C, normal marrow control, 1–6, samples from different pediatric patients. High molecular weight bands in lines 3 and 5 represent a normal NRAS band and a mutant band. Lines 1, 2, 4 and 6 represent wild type NRAS.

Single-strand conformation polymorphism (SSCP) was performed to PCR product to detect mutations. Products were mixed with 10 volumes of loading buffer, quenched on ice immediately, and applied to 5% polyacrylamide gel electrophoresis at 50 V, overnight, stained by silver nitrates and wrapped in plastic foil. Normal gene exhibits a specific conformational pattern, while mutant gene displays pattern with different electrophoretic mobility (mobility shift) which was confirmed by repeated SSCP (Figure 1).

## Statistical Methods

Data were processed and analyzed using SPSS for windows version 16.0 (SPSS, Inc, Chicago, IL, USA). Qualitative data were expressed as frequency and percentage and quantitative data were expressed as median. Chi-square test was used for comparative analysis. The prevalence of NRAS mutations in AML was too low to permit statistical analysis for correlation with survival. Kaplan-Meier analysis was used for survival of patients. The prognostic significance of the clinical variables was assessed using the Cox proportional hazards model. For all analyses, the *P* values were two-tailed, and a *P* value of less than 0.05 was considered statistically significant.

## Results

Mutant band in addition to wild bands were found in 6 (15.4%) of 39 pediatric AML patients, whereas 33 had only the NRAS wild-type allele (NRAS*^wild^*). No apparent clinical or biologic characteristics were significantly different for the patients with NRAS gene mutations when compared with those without NRAS mutations except that there was lower peripheral and bone marrow blast (P=0.01, P=0.04) in patients with NRAS mutation when compared with those without mutation (Table 1).

Patients were assigned to the following cytogenetic groups: t(8;21) (15.4%; 6/39), inv(16) (15.4%6/39), del 5 (5.1%;2/39), del 7 (2.6%;1/39); and with CN-AML (53.8%; 21/39). In 7.7% (3/39) of the cases, conventional karyotyping was not available (Table 2). Patients with Inv(16) is presented with highest WBC count(median 41 ×10^3^/μL), and t(8;21)AML patients with lowest WBCs (median 18 ×10^3^/μL) compared with the other cytogenetic groups (Table 2). The median ages of children with Inv (16) AML (7 years), with t(8;21) (8.3 years), with del(7) (8.5), and with del 5(8.9 years) were younger compared with CN-AML (9.5 years).

In the FAB subtype M4e, NRAS mutations were represented more frequently (50%, 3 of 6) than they were in all other subtypes. In the M3, M5, M6 and M7 subtypes, no NRAS mutation was detected, making NRAS mutations highly underrepresented in these subtypes. In all other FAB subtypes, the distribution of NRAS mutations did not differ significantly from each other. A detailed distribution of NRAS mutations in the respective FAB subtypes is presented in Table 3.

Based on cytogenetic findings, the 39 patients were segregated into three groups: a good-risk group (n =12) was defined by karyotype, t(8;21) or inv(16); a poor-risk group (n =3) by del(5) or del(7) and standard-risk group (n =21) by normal karyotypes.

Based on the previous findings, we analyzed the influence of NRAS mutations on the prognosis of pediatric AML patients for whom clinical follow up data were available. Table 4 shows the clinical outcome in pediatric AML patients. In the total group, there was no difference with regard to CR rate (NRAS^mutant^, 66.7%; NRAS^wild^, 69.5%; P = 0.43). Relapse was significantly more frequent in the AML patients with NRAS gene mutations (NRAS^muant^, 33.4 %; NRAS^wild^, 30.2 %; P= 0.26).

**Figure 2:**
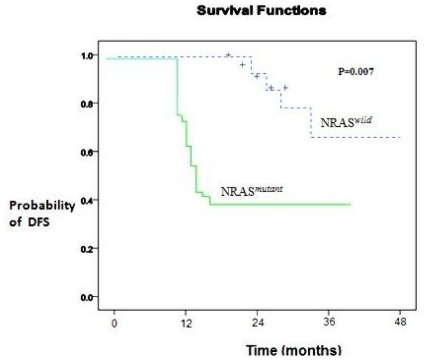
DFS in children with AML according to NRAS mutation.

**Figure 3:**
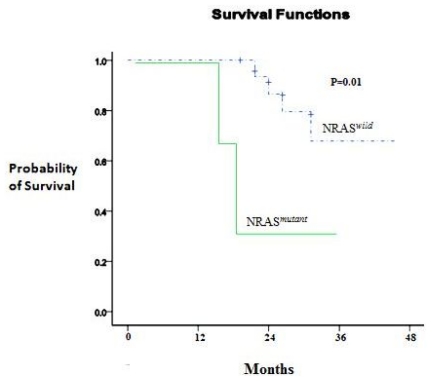
OS according to NRAS mutation.

At 3 years, the estimated OS was 30±15% in the presence of an NRAS mutation and 62±5% in its absence (P= 0.01). Adverse prognosis associated with N-ras mutations was observed in terms of 3 years DFS (NRAS*^mutant^* 35% vs NRAS*^wild^* 59%, P= 0.007), (Figure 2 and 3).

Univariate analysis showed that unfavorable prognostic factors for DFS were cytogenetic data (P = 0.005) and NRAS mutation (P = 0.002) (Table 5). Multivariate analysis showed NRAS was the strongest unfavorable factor (relative risk [RR], 3.6; P = 0.007), followed by cytogenetics (P = 0.02).

## Discussion

In recent years, a major focus of molecular cancer research has been the analysis of genes that may be causative in carcinogenesis (oncogenes). The clinical significance of RAS mutations has not been uniformly established. In the current study, we evaluated the clinical significance of NRAS mutations and investigated NRAS*^mutant^* by genomic PCR method in 39 newly diagnosed pediatric AML cases.

Activated RAS mutations confer proliferative and survival signals. Mutations in the NRAS gene are frequent genetic aberrations in adult AML.^19^ However, there have been only a few studies on childhood AML.^20^ With different mutation-detection techniques used and heterogeneous patient populations studied, the reported incidence of NRAS*^mutant^* in patients with childhood AML at presentation vary considerably; in our study 15.4% of pediatric AML patients (6/39) had NRAS*^mutant^*, corresponding to the reported frequency by others.^15^,^21^ Primary analyses revealed a statistically significant association between peripheral and bone marrow blast counts and NRAS mutation (P=0.01, P=0.04 respectively), however no significant differences had been found between the two groups with respect to age, gender, platelet count and WBCs count. These findings are in agreement with those reported in literatures.^10^,^13^

The highest frequency (33.3%) of NRAS mutations in our cohort (2/6) compared with the total cohort was detected in patients with inv(16). The high incidence of NRAS mutations in inv(16) in our study corresponded with most of the previously published studies reporting frequencies of 26% to 33% (22,23). 11q23/MLL aberrations are a frequent abnormality in pediatric AML.^24^,^25^,^26^ The frequency of 11q23/MLL-rearranged AML may have been underestimated because of low number of cases in the included study and because in our study as well as in other studies performed in the past, the cryptic MLL rearrangements may be not detected by conventional karyotyping. It is conceivable that the biological differences may lead to different treatment strategies for these age categories in the future.^27^ In our study, oldest children with AML were characterized by a high frequency of normal cytogenetic (53.8%) but the very young in the included study are characterized by higher frequency of inv (16).

The prognosis of AML depends on factors such as age, initial leukocyte count, FAB classification, karyotype, immune phenotype, and response to remission-induction therapy.^28^,^29^ Our study showed that cytogenetic was unfavorable prognostic factor among AML patients by univariate analysis. This was in agreement with other study who found that cytogenetic data is thought to be the most important prognostic factor for AML.^30^

The prognostic significance of NRAS mutation in both adults and children remains disputed. Generally NRAS gene mutation is associated with tumor progression and was reported to be associated with poor prognosis in solid tumors and acute lymphoblastic leukemia (ALL).^31^,^32^ Published reports addressing the clinical significance of NRAS mutations in patients with acute myeloid leukemia are inconclusive. Whereas some studies demonstrated a beneficial clinical effect of NRAS mutations,^33^,^34^ others reached a different conclusion (e.g. lower CR).^35^ Other studies also did not that show that patients with NRAS mutations had significantly good outcomes.^36^,^37^

In this study, the presence of NRAS gene mutation was related to similar complete remission (CR) rates following induction chemotherapy compared with non-mutated patients (66.7% vs. 69.5%, P=0.43). Those in CR had a similar relapse rate regardless of the presence of NRAS mutations (RR 33.4% vs. 30.2%, P=0.26). However, the presence of NRAS mutations was associated with poor three years OS and DFS compared with wild type cases (OS, P=0.01; DFS, P=0.007). This discrepancy between these studies findings and our study may be explained by differences in the intensity of the chemotherapy protocols employed to treat this group of patients and the small number of our cases.

## Conclusions

In addition to the evidence that activation of the RAS-signaling cascade contributes to the molecular pathogenesis of myeloproliferative disorders (38), NRAS mutation has adverse prognostic impact but further pediatric studies will be necessary to extend our knowledge and more precisely define the prognostic significance of NRAS mutations. This study also demonstrates the need to screen for specific translocation partners to allow appropriate treatment stratification like WT1 and FLT3.

## Figures and Tables

**Table 1:** NRAS mutations and clinical characteristics in pediatric AML cases

	No RAS mutation (n=33)	RAS mutation (n=6)	P
Age(median)	6,5	9,5	0,82
Leukocyte ×10^3^/μL (median)	40,1	42,5	0,92
Bone marrow blast%(median)	70,2	58	0,04
Peripheral blast% (median)	55,4	34	0,01

**Table 2:** Clinical characteristics according to cytogenetic aberrations

Cytogenetic	Frequency	Age(year)	WBCs (x10^3^/μL)
N=39	N. (%)	median	Median
t(8;21)	6 (15.4%)	8.3	18
inv(16)	6 (15.4%)	7.0	41
del(5)	2 (5.1%)	8.9	34
del(7)	1 (2.6%)	8.5	32
CN	21(53.8%)	9.5	38
Not done	3 (7.7%)	9.0	29

CN: cytogenetic normal

**Table 3:** Frequency of NRAS mutation according to FAB and cytogenetics

Cytogenetic	RAS mutation (n= 6)	NO RAS mutation (n= 33)

**Good-risk group**		
t(8;21)	1	5
t(inv16)	2	4

**Poor-risk group**		
del(5)	0	2
del(7)	0	1

**Normal group**		
normal karyotype	3	18
Not done	0	3

***FAB***		
M1	1	8
M2	1	11
M3	0	2
M4	1	4
M4e	3	3
M5	0	2
M6	0	1
M7	0	2

**Table 4:** NRAS mutation and clinical outcome

Patients	No RAS mutation	RAS mutation	P
All (n=39)			
CR(%)	69.5	66.7	0.43
RR(%)	30.2	33.4	0.26
3-year OS(%)	62	30	0.01
3-year DFS(%)	59	35	0.007

**Table 5:** Unvafourable prognostic factors for 3-year DFS in pediatric AML who achieved CR

Prognostic factors	Univariate	Multivariate		
	P value	P value	RR	95% CI

Age (years)				
10 years or older	0.3	0.2	2.1	1.2–6.5

FAB				
Other than M7	0.1	0.3	3.2	1.6–5.4

^*^Cytogenetic				
	0.005	0.02	2.4	0.97–6.2

NRAS mutation				
	0.002	0.007	3.6	1.5–8.5
